# Supplementary material for: Intrinsic protein disorder reveals divergent signaling architectures and lifestyle adaptation in Entamoeba species
Source: bioRxiv. 2026 Aug 12:2026.08.12.744381. Preprint. [Version 1] doi: 10.64898/2026.08.12.744381 (PMC13640752; doi:10.64898/2026.08.12.744381)
Supplement: Supplementary file 1 [file NIHPP2026.08.12.744381v1-supplement-1.pdf]

bioRxiv preprint doi: https://doi.org/10.64898/2026.08.12.744381; this version posted August 12, 2026. The copyright holder for this preprint (which was not certified by peer review) is the author/funder, who has granted bioRxiv a license to display the preprint in perpetuity. It is made available under a CC-BY 4.0 International license.

Supporting Information

Supplementary File 1. Excel file containing the complete protein datasets for all five species analyzed. For each protein, the table includes accession number, protein description, and predicted intrinsic disorder scores obtained using Disopred3 [27], IUPred3 [28], and AUCpreD [29]. Functional annotation is provided through associated Gene Ontology (GO) terms for Cellular Component, Molecular Function, and Biological Process. Enzyme Commission (EC) numbers are included where applicable.

Supplementary File 2. Excel file containing the top 10 enriched Gene Ontology (GO) terms for Cellular Component, Molecular Function, and Biological Process categories for each of the five

bioRxiv preprint doi: https://doi.org/10.64898/2026.08.12.744381; this version posted August 12, 2026. The copyright holder for this preprint (which was not certified by peer review) is the author/funder, who has granted bioRxiv a license to display the preprint in perpetuity. It is made available under a CC-BY 4.0 International license.

species analyzed. Enrichment results are stratified by predicted intrinsic disorder levels of proteins (0–10%, 10–30%, 30–70%, or >70%), with terms ranked by enrichment significance within each disorder bin for each species.
